# A single intraperitoneal injection of endotoxin in rats induces long-lasting modifications in behavior and brain protein levels of TNF-α and IL-18

**DOI:** 10.1186/1742-2094-9-101

**Published:** 2012-05-29

**Authors:** Paola Bossù, Debora Cutuli, Ilaria Palladino, Paola Caporali, Francesco Angelucci, Daniela Laricchiuta, Francesca Gelfo, Paola De Bartolo, Carlo Caltagirone, Laura Petrosini

**Affiliations:** 1 Clinical and Behavioral Neurology, IRCCS Fondazione Santa Lucia, Via Ardeatina 306, 00179, Rome, Italy; 2 Centro Europeo per la Ricerca sul Cervello (CERC)/Fondazione Santa Lucia, Via del Fosso di Fiorano 64, 00143, Rome, Italy; 3 Dipartimento di Psicologia, Università “Sapienza”, Via dei Marsi 78, 00185, Rome, Italy

**Keywords:** Neuroinflammation, Lipopolysaccharide (LPS), Behavioral impairment, Cytokines, Hippocampus, Frontal cortex, Cerebellum

## Abstract

****Background**:**

Systemic inflammation might cause neuronal damage and sustain neurodegenerative diseases and behavior impairment, with the participation of pro-inflammatory cytokines, like tumor necrosis factor (TNF)-α and interleukin (IL)-18. However, the potential contribution of these cytokines to behavioral impairment in the long-term period has not been fully investigated.

****Methods**:**

Wistar rats were treated with a single intraperitoneal injection of LPS (5 mg/kg) or vehicle. After 7 days and 10 months, the animal behavior was evaluated by testing specific cognitive functions, as mnesic, discriminative, and attentional functions, as well as anxiety levels. Contextually, TNF-α and IL-18 protein levels were measured by ELISA in defined brain regions (that is, frontal cortex, hippocampus, striatum, cerebellum, and hypothalamus).

****Results**:**

Behavioral testing demonstrated a specific and persistent cognitive impairment characterized by marked deficits in reacting to environment modifications, possibly linked to reduced motivational or attentional deficits. Concomitantly, LPS induced a TNF-α increase in the hippocampus and frontal cortex (from 7 days onward) and cerebellum (only at 10 months). Interestingly, LPS treatment enhanced IL-18 expression in these same areas only at 10 months after injection.

****Conclusions**:**

Overall, these results indicate that the chronic neuroinflammatory network elicited by systemic inflammation involves a persistent participation of TNF-α accompanied by a differently regulated contribution of IL-18. This leads to speculation that, though with still unclear mechanisms, both cytokines might take part in long-lasting modifications of brain functions, including behavioral alteration.

## Background

The impact of systemic inflammation on the pathogenesis of chronic brain diseases, such as neurodegenerative diseases, is a current and challenging topic of biomedical research [1,2]. Pro-inflammatory cytokines, which have effects on both immune and nervous systems, are central players of inflammation and may contribute to brain changes during both physiological and pathological processes. As key mediators of a chronic neuroinflammation that drives progressive tissue damage in the brain, cytokines could take part to the pathogenesis and progression of neurodegenerative diseases [3-5]. However, a better understanding of brain cytokine role in behavioral and cognitive performances following systemic inflammation, especially in the longer term after inflammatory insults, remains an important area of research. Peripheral injection of the bacterial endotoxin component lipopolysaccharide (LPS) that models systemic infection, has been widely used to induce neuroinflammation, since it results in the early brain synthesis of inflammatory cytokines, such as interleukin (IL)-1β, IL-6, and tumor necrosis factor (TNF)-α [6-8]. These inflammatory mediators, in turn, appear central in driving behavioral modifications, as in the case of the behavioral responses to LPS, known as sickness behavior, which is the acute consequence of cytokine elevation [9]. Notably, endotoxin triggering can also induce protracted behavioral effects, as occurring in aging rats [10,11], as well as in animal models of prion-disease [12,13] and amyotrophic lateral sclerosis [14], where progression of symptoms and neurodegenerative processes are persistently exacerbated after LPS treatment.

Differently, investigations in healthy adults aimed at determining whether a single event of systemic inflammation can as such induce long-lasting modifications of behavior and brain cytokine synthesis are still limited. Remarkably, two studies have been previously addressed to investigate this issue in rodent models. A single LPS administration results in delayed loss of neurons and cholinergic innervations, leading to enduring behavioral alterations characterized by memory deficits and changes in exploratory patterns [15]. Moreover, TNF-α appears to convey inflammation from periphery to brain, where it is early and persistently released after LPS treatment and causes microglia activation and significant loss of neurons between 7 and 10 post-treatment months [16]. Thus, it is predictable that additional cytokines could take part to neuroinflammatory phenomena leading to chronic progression of neurodegeneration. Notably, the pro-inflammatory cytokine IL-18 has recently emerged as a key player in neuroinflammatory and neurodegenerative processes, with wide behavioral and cognitive effects [17-19]. However, the time-dependent modifications of IL-18 expression within specific brain regions after a peripheral neuroinflammatory event have not yet been delineated.

The present research is aimed at characterizing the long-term consequences of systemic inflammation on behavioral/cognitive performances and brain cytokine changes. To this aim, analyses of motivational, spatial, mnesic, discriminative, and attentional functions, as well as regional brain level measurements of the two cytokines TNF and IL-18 were performed at 7 days and 10 months after a single intraperitoneal injection of high-dose LPS.

## Methods

### **Experimental design**

**Figure 1:**
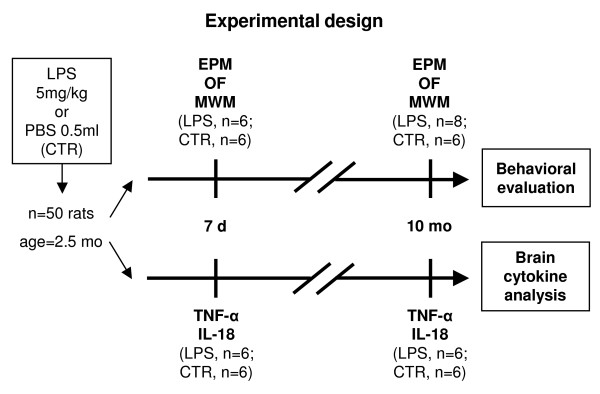
**Experimental design.** Fifty adult male Wistar rats were intraperitoneally injected with either LPS (5 mg/kg in 0.5 mL) or PBS (0.5 mL). The 26 animals subjected to behavioral evaluations were divided into two groups (LPS, *n* = 14; CTR, *n* = 12) which were used for Elevated Plus Maze (EPM), Open Field with Objects (OF), and Morris Water Maze (MWM) assessments, respectively, at 7 days and 10 months following LPS or PBS injections. The remaining 24 animals that underwent brain cytokine analyses were divided into two groups (LPS, *n* = 12; CTR, *n* = 12) for the measurement of regional TNF-α and IL-18 levels at 7 days and 10 months following LPS or PBS injections.

Fifty adult male Wistar rats (weighing 200 g at the beginning of the experiments; Harlan, Italy) were used. The animals were pair-housed and kept under standard conditions with food and water *ad libitum* on a 12/12 h dark/light cycle (light on between 07:00 and 19:00 h). The animals belonging to the LPS-treated group (LPS, *n* = 26) were intraperitoneally (i.p.) injected with a single dose of LPS from E. coli, strain 055:B5 (5 mg/kg; Sigma, Italy) in 0.5 mL of volume, according to Qin and colleagues [16]. The animals belonging to the control group (CTR, *n* = 24) were i.p. injected with 0.5 mL of vehicle (sterile endotoxin-free PBS, Life Technologies, Italy). To avoid potential influence of behavioral testing on cytokine levels, cytokine assessment and behavioral testing were performed on different animals. Thus, 26 rats (LPS, *n* = 14; CTR, *n* = 12) were subjected to behavioral evaluations, while 24 animals (LPS, *n* = 12; CTR, *n* = 12) underwent brain cytokine analyses (Figure 1). Behavioral testing and cytokine analyses were performed after LPS challenge at two time-points, representative of long-term responses in its early (7 days) and late (10 months) phases.

Following i.p. injections, animals were observed for the first 4 h and the presence of sickness behavior was evaluated in all animals by measuring changes in body weight and temperature. In addition, presence of piloerection and lethargy was observed for 1 min at five post-treatment time-points (0, 1, 2, 3, and 4 h). LPS injections induced significant body weight loss (one-way ANOVA: F_1,48_ = 7.1, *P* <0.01) and body temperature increase (F_1,48_ = 4.01, *P* <0.05). The number of animals showing lethargy and piloerection progressively increased as the LPS post-injection hours went by, as indicated by Chi-square test. Namely, in LPS animals the presence of piloerection became significant from the first hour onward and the presence of lethargy from the second hour onward (*χ*^2^ = 6.0; d.f. = 1; *P* <0.01).

According to other authors [16], an increased level of serum TNF-α was observed in treated animals at 4 h after LPS treatment (186.6 ± 37.5 pg/mL; mean ± SE), whereas in CTR rats this cytokine level was low and undetectable under our experimental conditions. At difference, serum IL-18 was undetectable in both LPS and CTR animals.

### **Behavioral assessment**

The behavioral assessment lasted two days and consisted of three tests: Elevated Plus Maze, Open Field with Objects, and Morris Water Maze.

#### ***Elevated plus maze (EPM)***

The EPM raised 90 cm above the ground was formed by a wooden structure in the shape of a cross with four arms (50 cm × 10 cm). The north and south arms were opened, while the east and west arms were enclosed by 36-cm-high walls. The rat placed on the central platform was allowed to explore the maze for 5 min. The total time spent in the open and close arms was measured.

#### ***Open field with objects (OF)***

The apparatus consisted of a circular arena (diameter 140 cm) delimited by a 30-cm-high wall. During session 1 (S1), each rat was allowed to freely move in the empty open field and its baseline activity level was measured. During the habituation phase (S2-S4), four objects were placed in a square arrangement in the middle annulus of the arena and a fifth one was placed in the central area. The five objects were: (1) a metal bar with a conical base; (2) a plunger; (3) a long steel rod; (4) a yellow rubber plug; and (5) a black cylinder with a plastic cup turned upside down on top of it. During the spatial change (S5 and S6), the spatial configuration was changed by moving objects 2 and 5 so that the initial square arrangement was modified to a polygon-shaped configuration, without any central object. During the novelty session (S7), the configuration was modified by substituting object 4 with a green plastic object shaped like a half moon. All sessions lasted 6 min; inter-session intervals lasted 3 min. Rats’ behavior was recorded by a video camera whose signal was relayed to a monitor and to an image analyzer (Ethovision, Noldus, Wageningen, The Netherlands).

In S1 the following motor and emotional parameters were analyzed: total distance (in cm) traveled in the arena; percentage of distances traveled in a 20-cm peripheral annulus; number of central crossings; motionless time; number of defecation boluses. In S2-S4 total time spent in contacting object was analyzed. Contact was considered to have taken place when the rat’s snout actually touched an object or when it sniffed the object for at least 1 s, but not when the rat leaned against, stood, or sat on the object. In S5-S7 the time spent contacting objects was expressed as discrimination index (d index), that is time exploring displaced (or novel) objects minus time exploring not displaced (or familiar) objects/total exploration time.

#### ***Morris water maze (MWM)***

The rats were placed in a circular white pool (diameter 140 cm) filled with 24°C water made opaque by the addition of a-toxic acrylic color (Giotto, Italy). An escape platform (diameter 10 cm) was submerged 2 cm below the water level. Each rat was submitted to two consecutive phases (Place and Probe) consisting of eight trials (Place) and one trial (Probe). During Place, the rat released into the water from randomly varied starting points was allowed to find the hidden platform for a maximum of 120 s. When the rat reached the platform, it was allowed to remain there for 30 s. The inter-trial interval was 3 min. Then, the platform was removed and rats were allowed to swim for 60 s in searching for it (Probe phase). Rats’ navigational trajectories were recorded by a video camera whose signal was relayed to a monitor and to the previously described image analyzer. In analyzing MWM performances the following behavioral parameters were considered: latencies to find the platform; total distances swum in the pool; mean swimming velocity; percentage of time spent in the rewarded quadrant (presence of platform) in the Probe phase; navigational strategies put into action in reaching the platform. The navigational strategies were classified in five main categories [20], regardless the platform was reached or not: Circling (C), that is, swimming in a 20-cm peripheral annulus, with inversion of swimming direction and counterclockwise and clockwise turnings in the peripheral sector of the pool; Extended Searching (ES), that is, swimming around the pool in all quadrants, visiting the same areas more than once; Restricted Searching (RS), that is, swimming in some pool quadrants, not visiting some tank areas at all; Restricted Circling (RC), that is, reaching the platform by swimming only in the peripheral annulus; direct Finding (F), that is, swimming towards the platform without any foraging around the pool. Two researchers who were unaware of the individual specimen’s group categorized the swimming trajectories drawn by the image analyzer. They attributed the dominant behavior in each trial to a specific category. Categorization was considered reliable only when their judgments were consistent.

### **Assessment of protein levels of cytokines in serum and brain tissue**

For serum cytokine measurement, blood samples were taken in terminally anaesthetized rats and collected in microfuge tubes. Samples were spun down and serum kept at −80°C until further use.

For brain cytokine measurement, rats were sacrificed by decapitation and the brains were quickly removed and dissected on ice using a binocular dissection microscope. Frontal cortex, hippocampus, striatum, cerebellum, and hypothalamus were dissected on ice by a trained researcher according to Glowinski and Iversen’s method [21]. All brain regions were extracted in 1 mL extraction buffer/100 mg tissue. Brain tissue samples were homogenized in an ice-cold lysis buffer containing 137 mM NaCl, 20 mM Tris–HCl (pH 8.0), 1% NP40, 10% glycerol, 1 mM PMSF 10 μg/mL aprotinin, 1 μg/mL leupetin, and 0.5 mM sodium vanadate. The tissue homogenate solutions were centrifuged with 14000 × *g* for 25 min at 4°C. The supernatants were collected and stored at −80°C until analysis.

Quantification of serum and intracerebral TNF-α and IL-18 protein levels were assessed by ELISA kit (Biosource, Invitrogen), according to manufacturer’s instructions. The limit detection of assays was 4 pg/mL for TNF-α and 15 pg/mL for IL-18. Cytokine results, reported as picograms of the measured molecule per mL of serum (pg/mL) or per gram of tissue (pg/g) are expressed as mean values ± SEM. Where indicated, cytokine amounts were also normalized to protein content. In this case, the concentration of total protein in the brain extracts was measured by Bradford assay (BioRad Laboratories).

### **Statistical analysis**

The data were firstly tested for normality (Wilk-Shapiro’s test) and homoscedasticity (Levene’s test). Then, they were analyzed by one-way or two-way ANOVAs for independent (treatment, time) and repeated (trial, session, arm) measures followed by Tukey’s HSD test. Non-parametric data related to piloerection and lethargy evaluation were analyzed by means of a Chi-squared metric. The significance level was established at *P* <0.05.

### Ethical approval

The experimental research reported in this manuscript has been performed with the approval of the Ethical Committee on animal experiments of Fondazione Santa Lucia and all efforts were made to minimize animal suffering and to reduce their number, in accordance with the European Community Council Directive of 24 November 1986 (86/609/EEC).

## Results

### **Seven days following LPS treatment**

#### ***EPM***

**Figure 2:**
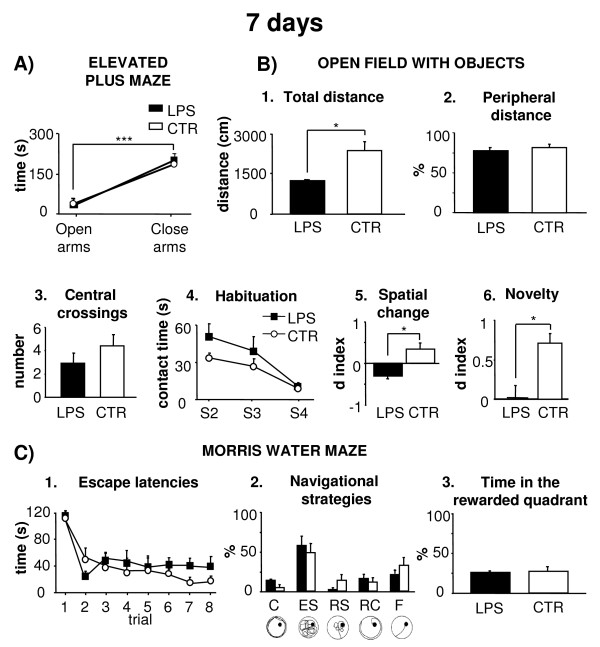
**Effects of i.p. injection of LPS on behavioral performances at 7 days post-treatment.** (**A**) Mean time spent in the open and close arms by the animals in Elevated Plus Maze is depicted. (**B**) Total distance (1), peripheral distance (2), central crossings (3), mean contact times with objects during habituation phase (S2-S4) (4), spatial change (5), and novelty (6) exhibited by the two experimental groups in Open Field with Objects are depicted. (**C**) Mean escape latencies to reach the platform (1), navigational strategies (2), and time spent in the rewarded quadrant (3) displayed by the two experimental groups in Morris Water Maze are depicted. In this figure, the circular figurines illustrate the typical explorative patterns of the five navigational strategies (C, Circling; ES, Extended Searching; RS, Restricted Searching; RC, Restricted Circling; F, direct Finding). Asterisks inside the graphs indicate the significance of comparisons between groups: * *P* <0.05, *** *P* ≤0.001.

No differences in anxiety levels were evident between groups (Figure 2A). A two-way ANOVA (treatment x arm) revealed that all animals spent more time in the close *vs.* open arms (arm effect: F_1,10_ = 141.68, *P* <0.001), without significant treatment effect (F_1,10_ = 0.31, *P* n.s.) and interaction (F_1,10_ = 0.59, *P* n.s.).

#### ***OF***

In S1, LPS rats traveled around the arena significantly less in comparison to CTR (Figure 2, B1), while no significant treatment effect was evident on peripheral distances and on the number of central crossings (one-way ANOVAs: total distance: F_1,10_ = 5.37, *P* <0.05; peripheral distance: F_1,10_ = 0.86, *P* n.s.; central crossings: F_1,10_ = 1.32, *P* n.s.) (Figure 2, B2-3). All animals exhibited comparable levels of anxiety as indicated by the absence of significant differences between groups in the number of defecation boluses and motionless time (defecation boluses: F_1,10_ = 0.01, *P* n.s.; motionless time: F_1,10_ = 1.52, *P* n.s).

In S2-S4, all animals showed habituation, progressively decreasing exploration time of objects (Figure 2, B4). A two-way ANOVA (treatment x session) revealed a significant session effect (F_2,20_ = 9.93, *P <*0.05). Treatment and interaction effects were not significant (treatment: F_1,10_ = 4.38, *P* n.s.; treatment x session: F_2,20_ = 0.60, *P* n.s.).

In S5, while CTR rats detected the new spatial arrangement and contacted displaced objects more than non-displaced objects, LPS rats failed to detect the new spatial arrangement (one-way ANOVA: F_1,10_ = 6.60, *P* <0.05) (Figure 2, B5). In S6, while CTR rats no more reacted to the spatial change, LPS rats persisted in not reacting to change (F_1,10_ = 0.91, *P* n.s.).

In S7, LPS rats failed to recognize the novel object. Significant treatment effect was found on d index (F_1,10_ = 6.59, *P* <0.05) (Figure 2, B6).

#### ***MWM***

*Place***.** No significant differences in reaching the hidden platform were evident between experimental groups (Figure 2, C1-2). A two-way ANOVA (treatment x trial) confirmed that all rats displayed a progressive latency reduction as trials went by (F_7,70_ = 15.47, *P* <0.001), without significant treatment effect (F_1,10_ = 1.30, *P* n.s.), and interaction (F_7,70_ = 2.15, *P* n.s.). No differences were found in the swimming velocities (LPS rats $\bar{\text{x}}$ : 21.70 ± 0.53 cm/s; CTR rats $\bar{\text{x}}$ : 21.48 ± 2.44 cm/s; one-way ANOVA: F_1,10_ = 0.79, *P* n.s.) as well as in the total distances (two-way ANOVA: treatment effect: F_1,10_ = 0.81, *P* n.s.; trial effect: F_7,70_ = 30.09, *P* <0.001; interaction: F_7,70_ = 2.01, *P* n.s.). The analysis of the navigational strategies did not reveal any difference between groups.

*Probe***.** No relevant differences were evident between experimental groups, as revealed by a one-way ANOVA on the percentage of time spent in the previously rewarded quadrant (F_1,10_ = 0.07, *P* n.s.) (Figure 2, C3).

#### ***Cytokine analysis***

**Figure 3:**
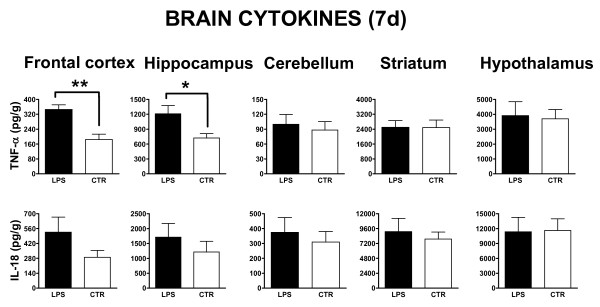
**Effects of i.p. injection of LPS on brain regional expression of cytokines at 7 days post-treatment.** The levels of TNF-α (upper row of panels) and IL-18 (lower row of panels) in LPS-treated (LPS, black bar) and PBS injected control animals (CTR, open bar) were assessed 7 days post-treatment by using specific ELISA, as described in the Materials and methods section. Results are reported as histograms representing the cytokine mean concentrations with SEM error bars for each dissected brain region. Values measured from each group of rats (*n* = 6) were calibrated by tissue weight. Asterisks inside the graphs indicate the significance of comparisons between groups: * *P* <0.05, ** *P* <0.01.

Figure 3 depicts the levels of TNF-α and IL-18 in frontal cortex, hippocampus, cerebellum, striatum and hypothalamus measured 7 days after LPS or PBS injections.

With regard to TNF-α (Figure 3, upper panels), its levels were significantly higher in LPS *vs.* CTR animals in hippocampus (one-way ANOVA: F_1,10_ = 6.81, *P* <0.05) and in the frontal cortex (F_1,10_ = 17.12, *P* <0.01). No significant differences in TNF-α levels were found between LPS and control animals in cerebellum, striatum, and hypothalamus.

IL-18 levels (Figure 3, lower panels) were in general slightly more elevated in most brain regions of LPS, as compared to CTR animals, but at variance with TNF-α, the statistical significance was not reached in any of the analyzed cerebral regions. However, a trend towards IL-18 increase for LPS as compared to CTR rats was observed in frontal cortex (526.0 ± 141.6 *vs.* 290.3 ± 64.73; *P* = 0.16). In this experiment, cytokine data have been also calculated by normalizing to protein content instead than to tissue weight and very similar results have been obtained with the two methods (not shown). No detectable TNF-α and IL-18 levels have been found in serum of both LPS and CTR animals.

### **Ten months following LPS treatment**

#### ***EPM***

**Figure 4:**
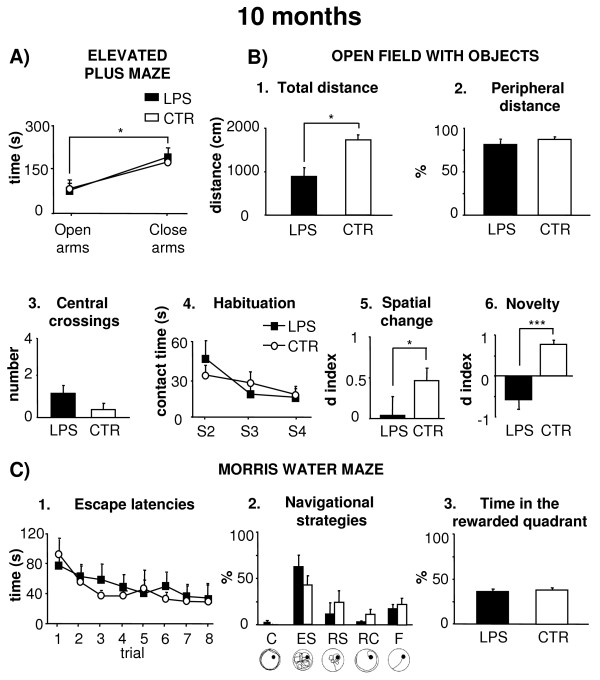
**Effects of i.p. injection of LPS on behavioral performances at 10 months post-treatment.** (**A**) Mean time spent in the open and close arms by the animals in Elevated Plus Maze is depicted. (**B**) Total distance (1), peripheral distance (2), central crossings (3), mean contact times with objects during habituation phase (S2-S4) (4), spatial change (5), and novelty (6) exhibited by the two experimental groups in Open Field with Objects are depicted. (**C**) Mean escape latencies to reach the platform (1), navigational strategies (2), and time spent in the rewarded quadrant (3) displayed by the two experimental groups in Morris Water Maze are depicted. In this figure, the circular figurines illustrate the typical explorative patterns of the five navigational strategies (C, Circling; ES, Extended Searching; RS, Restricted Searching; RC, Restricted Circling; F, direct Finding). Asterisks inside the graphs indicate the significance of comparisons between groups: * *P* <0.05, *** *P* ≤0.001.

The two experimental groups did not differ on anxiety levels and all animals exhibited the standard open arms avoidance (Figure 4A). A two-way ANOVA (treatment x arm) indicated not significant treatment effect (F_1,12_ = 0.59, *P* n.s.) and interaction (F_1,12_ = 0.10, *P* n.s.), while arm effect was significant (F_1,12_ = 6.91, *P* <0.05).

#### ***OF***

In S1, LPS rats traveled around the arena significantly less in comparison to CTR animals (Figure 4, B1), although significant differences between groups were not evident on peripheral distances and number of central crossings (one-way ANOVAs: total distance: F_1,12_ = 11.45, *P* <0.05; peripheral distance: F_1,12_ = 1.77, *P* n.s.; central crossings: F_1,12_ = 2.78, *P* n.s.) (Figure 4, B2-3). All animals exhibited comparable levels of anxiety as indicated by the absence of significant differences between groups in the number of defecation boluses and motionless time (defecation boluses: F_1,12_ = 0.58, *P* n.s.; motionless time: F_1,12_ = 2.47, *P* n.s).

All animals habituated as the total amount of time spent exploring the five objects decreased from S2 to S4 (session effect: F_2,24_ = 12.33, *P* < 0.001) (Figure 4, B4). Treatment effect (F_1,12_ = 0.12, *P* n.s.) and interaction (F_2,24_ = 0.31, *P* n.s.) were not significant.

In S5, differently from CTR, LPS animals did not detect the spatial change (one-way ANOVA: F_1,12_ = 5.39, *P <* 0.05) (Figure 4, B5). In S6, LPS rats did not still react to the spatial change, while CTR animals stopped to react to change (F_1,12_ = 0.71, *P* n.s.). Once more, in S7, when the novel object was placed in the arena, LPS rats did not recognize it (F_1,12_ = 21.65, *P* <0.001) (Figure 4, B6).

#### ***MWM***

*Place***.** No significant differences in reaching the hidden platform were evident between groups (Figure 4, C1-2). A two-way ANOVA (treatment x trial) revealed that both groups displayed a progressive latency reduction as trials went by (F_7,84_ = 7.02, *P* <0.001), without significant treatment effect (F_1,12_ = 0.02, *P* n.s.) and interaction (F_7,84_ = 1.21, *P* n.s.). No differences were found in the swimming velocities (LPS $\bar{\text{x}}$ : 19.73 ± 0.75 cm/s; CTR $\bar{\text{x}}$ : 18.99 ± 1.46 cm/s; one-way ANOVA: F_1,12_ = 0.17, *P* n.s.) as well as in the total distances (two-way ANOVA: treatment effect: F_1,12_ = 0.99, *P* n.s.; trial effect: F_7,84_ = 4.84, *P* <0.001; interaction: F_7,84_ = 0.38, *P* n.s.). Furthermore, no significant differences between groups were observed on the navigational strategies.

*Probe***.** A one-way ANOVA on the percentage of time spent in the previously rewarded quadrant failed to reveal any significant treatment effect (F_1,12_ = 0.20, *P* n.s.) (Figure 4, C3).

#### ***Cytokine analysis***

**Figure 5:**
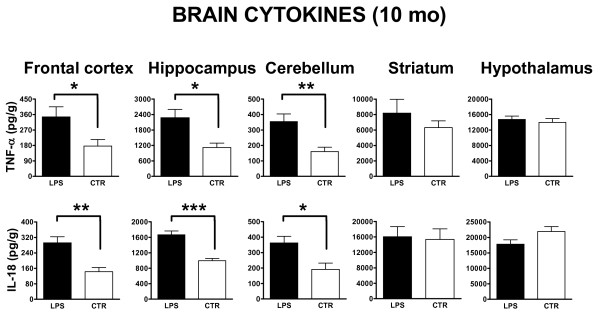
**Effects of i.p. injection of LPS on brain regional expression of cytokines at 10 months post treatment.** The levels of TNF-α (upper row of panels) and IL-18 (lower row of panels) in LPS-treated (LPS, black bar) and PBS-injected control animals (CTR, open bar) were assessed 10 months post-treatment by using specific ELISA, as described in the Materials and methods section. Results are reported as histograms representing the cytokine mean concentrations with SEM error bars for each dissected brain region. Values measured from each group of rats (*n* = 6) were calibrated by tissue weight. Asterisks inside the graphs indicate the significance of comparisons between groups: * *P* <0.05, ** *P* <0.01, *** *P* <0.001.

The increase of TNF-α already observed in the earlier post-treatment stage was maintained in the brain regions of LPS *versus* CTR animals (Figure 5, upper panels). In particular, the significant increase of TNF-α was maintained in the frontal cortex (one-way ANOVA: F_1,10_ = 8.16, *P* <0.05) and hippocampus (F_1,10_ = 9.91, *P* <0.05) and reached in cerebellum (F_1,10_ = 11.86, *P* = 0.01). Furthermore, not significant differences in TNF-α levels were observed in striatum and hypothalamus.

Alike to TNF-α, IL-18 levels were significantly more elevated in LPS than CTR animals (Figure 5, lower panels) in frontal cortex (F_1,10_ = 15.31, *P*  <0.01), hippocampus (F_1,10_ =  33.46, *P* <0.001), and cerebellum (F_1,10_ = 8.49, *P* <0.05). Again, no significant changes in IL-18 levels occurred in striatum and hypothalamus.

At this time point serum TNF-α and IL-18 levels have not been evaluated.

## Discussion

Progressive neurodegeneration has been proposed to be driven by chronic persistent inflammation, but up to now the chronic neuroinflammatory processes potentially implicated in neuronal damage leading to cognitive impairment are scarcely detailed. In the present study we show that a single peripheral inflammatory insult induces persistent discriminative deficits accompanied by different regional patterns of brain TNF-α and IL-18 expression.

As early consequence of LPS treatment (7 days), we observed a motor hypoactivity and discriminative deficits (in OF) not associated to any spatial memory deficit (MWM Probe trial results) or anxiety expression (in EPM and OF). In particular, LPS rats exhibited marked discriminative deficits, when in OF spatial arrangement and saliency of the objects were changed. These results are in line with the impaired object recognition described in mice 4 days after LPS treatment [22].

These behavioral changes may be interpreted in terms of reduced motivation resulting in reduced responsivity toward the context. Noteworthy, reduced motivation leading to diminished investigation of juvenile conspecifics in the social exploration has been described in rodents at very early stage following LPS treatment [23,24]. Nevertheless, it has to be underlined that the lack of spatial change detection in LPS rats might be ascribed also to an attentional deficit disrupting some, although not all, components of the spatial information processing. This faceted behavioral pattern, already encountered in the presence of cholinergic depletion, could be linked to the different stress levels of MWM and OF tasks [25]. Actually, although both tasks are hippocampal-dependent, MWM puts forward attentional requests but involves high stress levels given the forced swimming in the search of the escape platform, while OF requires high attentional load but in an experimental context not closely related to survival and thus with lower stress levels. Thus, the different findings observed in MWM and OF tasks could be linked to the saliency of the MWM platform higher than the mere spatial re-arrangement of OF objects.

The late behavioral pattern (10 months) was similar to that displayed 7 days after LPS injection. While no modification of spatial memory or anxiety levels was observed, motor hypoactivity and discriminative deficits were once again present, indicating that a systemic inflammatory response evoked by a single LPS injection (5 mg/kg) is able to significantly affect cognitive functions even 10 months later. These results are in line with the report by Semmler and colleagues [15] that described behavioral and cognitive impairment, neuronal loss in frontal cortex and hippocampus, as well as reduced cholinergic innervation in parietal cortex occurring up to 3 months after treatment in a rat model of sepsis evoked by high-dose LPS (10 mg/kg). Furthermore, two intraperitoneal LPS injections (500 μg/kg) at postnatal days 7 and 9 in rat pups caused reduced locomotor activity and deficits in object recognition memory tested at day 70, as well as decreased expression of parvalbumin immunoreactive neurons in the CA1-CA3 hippocampal regions and no changes in frontal cortex [26].

It is to be considered that the explorative and discriminative deficits found in the present study could be also linked to a specific loss of dopaminergic neurons. In fact, in experimental conditions similar to those of our study, LPS progressively reduces the number of tyrosine hydroxylase-immunoreactive neurons in the substantia nigra [16]. Interestingly, bilateral lesions of the pars compacta of the substantia nigra elicit in rats impairment of object recognition associated with microglial activation in the hippocampus, but not in the striatum [27]. Taking into account these observations, the long-term impairment of exploratory and discriminative functions observed in our LPS rats could be related to a reduced functionality of either cholinergic [15] and dopaminergic [16] systems. These two systems appear to be implicated the former one in the attentional tuning controlling discriminative behaviors [28,29] and the latter one in the locomotory function and motivational drive to explore objects [30,31].

As previously described, at the time-points chosen as representative of early and late stages of long-term LPS response, the analysis of the behavioral performances has been matched by analysis of brain cytokine expression. Interestingly, LPS treatment was able to elevate the expression of TNF-α in definite brain regions across the entire experimental period. In particular, since our first evaluation (7 days), TNF-α was significantly elevated in the frontal cortex and in the hippocampus and it remained significantly higher than in CTR animals also at 10 months. In addition, TNF-α levels were increased even in the cerebellum 10 months after LPS treatment. These results are in line with a previous study, where 2 months after peripheral LPS administration, a TNF-α mRNA increase was observed in the cortex and hippocampus, as well as cerebellar structures [32].

The lack of neuroinflammatory reactions in the striatum could be ascribed to the fact that either specific neuronal subtypes and brain regions are susceptible to inflammation more than others in relation to microglial component [33,34]. Conversely, the unresponsiveness of the hypothalamus to LPS insult could be attributed to the late times analyzed in our study, given that after systemic LPS administration a transient increase in TNF-α, ranging from 1 to 3 to 18 h, has been previously observed [6,35,36].

Intriguingly, IL-18 levels significantly increased in frontal cortex, hippocampus, and cerebellum at the latest (10 months), but not at the earlier (7 days) stage of LPS-induced long-term neuroinflammation. On the other hand, the very low levels of both TNF-α and IL-18 we observed in serum of treated animals 7 days after the LPS treatment are in agreement with previous studies describing that the circulating levels of pro-inflammatory cytokines increase soon after the endotoxic insult, but that after the acute response they subside in the blood, while stay elevated in the brain [16]. Furthermore, these results strongly suggest that in this study, the measured TNF-α and IL-18 elevation in the brain is independent from their concentration in the blood.

Therefore, according to their profile of expression, the two cytokines seem to differently participate in brain inflammatory response. Regarding TNF-α, our results support and further expand the view that this cytokine is an early and persistent mediator of the long-lasting inflammatory cascade [16]. In fact, TNF-α might have a pivotal role in conveying inflammation from periphery to brain, as primary stimulus that promotes self-propelling mechanism of microglial activation, induces persistent brain production of pro-inflammatory cytokines including IL-1β and, ultimately, leads to progressive neurodegeneration. Differently from TNF-α, IL-18 might act as a delayed mediator of neuroinflammation, in agreement with data obtained in experimental models of brain ischemia and trauma [37,38].

Regarding the possible molecular mechanisms, while quite a lot of evidence corroborates the hypothesis of an involvement of brain TNF-α in neurodegeneration and cognitive decline, even identifying some of its driving mechanisms [16,39-43], much less data are available about the molecular links between brain IL-18 expression and cognitive functions.

In line with our data, IL-18 has been previously found constitutively expressed in the cortex, hippocampus, cerebellum, hypothalamus, and striatum [44] and it is also detectable in astrocytes and microglia after LPS treatment [45,46]. However, by means of immunocytochemistry analysis, other studies have shown, that IL-18 protein is mainly expressed in the neurons of the medial habenula and in the ependymal cells surrounding the lateral and the third ventricles, without significant staining in microglial cells or astrocytes [47]. Therefore, even though microglia might be a relevant cellular source of increased IL-18 in our experimental conditions, additional studies should be performed to identify the IL-18 producing cells within the brain in the presence of LPS-mediated long-term neuroinflammation. Although with some fluctuations, we observed the expression of IL-18 protein (both constitutive and LPS-induced) in the brains of the rats analyzed both at 7 days and at 10 months. These data are in agreement with other results showing that IL-18 is present in immature and adult brain, as the cytokine is present in neonatal brain in normal conditions and during hypoxic-ischemic brain injury [48,49] and it is involved in neuronal cell death during traumatic brain injury both in immature and adult brain [38,50]. In fact, although this cytokine has been generally associated with the amplification of age-dependent inflammatory processes [51] and it may be relevant in age-related functional impairment [52], its role in CNS might be important in both health and disease since the development, throughout the whole life.

In neuroinflammatory conditions, given that brain cytokines function as an integrated network [53], the increased expression of IL-18, probably modulated by TNF-α itself [54], could participate in perpetuating brain inflammation by stimulating the activation of the transcription factor NF-κB and the production of other pro-inflammatory mediators, such as IL-1β and TNF-α [55,56]. As a consequence, the increase of IL-18 expression in the brain might influence progressive neurodegeneration and cognitive dysfunction. In fact, in both experimental and clinical settings, IL-18 appears to directly or indirectly interfere with the processes of memory consolidation, synaptic plasticity and/or neurogenesis [18,57-60]. Noteworthy, the role of IL-18 in mediating the behavioral effects of LPS injection observed in our conditions is supported by a recent study showing that the expression of the IL-18 signaling receptor and of its short variant considered a potential negative regulator of IL-18, is tightly modulated by LPS and occurs in specific brain areas associated with the limbic system [61].

Overall, in spite of the intriguing association between behavioral/cognitive impairment and cytokine expression observed in the present research, we are aware that the present data do not allow defining the causal role of the increased TNF-α and IL-18 expression in behavioral and cognitive abnormalities. Similarly, they do not allow identifying at brain regional level the molecular mechanisms of chronic inflammatory network that could participate in long-term cognitive decline. Nonetheless, by taking together other authors’ results and ours, it is possible to hypothesize that the peripheral administration of high-dose LPS could cause TNF-α dependent activation of microglia and subsequent increase of brain cytokines, like TNF-α itself, IL-1β, and IL-18 [16], the present paper] which might concur in inducing persistent behavioral alterations [15], the present paper] probably caused by a long-term loss of neurons [15,16]. In addition, LPS-induced cytokine alterations may influence behavior by affecting neurotransmitterial function, neuroendocrine activity, neuronal plasticity properties and brain circuitry [62], mechanisms and functions whose evaluation is beyond the scope of the present study. Thus, whether and how a direct association between behavioral dysfunction and brain cytokine elevation indeed exists remains an open question.

## Conclusions

The present study describes a persistent behavioral impairment and a protracted and distinctive elevation of brain TNF-α and IL-18 expression, as long-term consequences of a single inflammatory insult occurred 10 months before. These observations appear to be important elements at the base of inflammation-induced cognitive/behavioral impairment, suggesting that both cytokines, although with a different pattern, might take part in the enduring deterioration of behavioral functions caused by systemic inflammation.

Furthermore, these findings support the recently hypothesized participation of IL-18 in the progressive neurodegeneration that occurs in some forms of dementia.

## Abbreviations

C: circling; CNS: central nervous system; CTR: control; EPM: Elevated Plus Maze; ELISA: Enzyme-linked immunosorbent assay; ES: Extended Searching; F: direct Finding; i.p.: intraperitoneally; IL: interleukin; LPS: lipopolysaccharide; MWM: Morris Water Maze; OF: Open Field with Objects; PBS: phosphate buffered saline; RC: Restricted Circling; RS: Restricted Searching; S1-7: session 1–7; TNF: tumor necrosis factor.

## Competing interests

The authors declare that they have no competing interests.

## Authors’ contribution

PB, FA, and LP designed the study. DC, PC, and DL treated animals and performed all behavioral evaluations. FA, FG, and PDB performed the dissection of brain areas. IP prepared brain samples and performed cytokine experiments. All authors analyzed and discussed the data. PB, DC, IP, and LP wrote the initial draft. All authors made contributions in writing and discussing the manuscript. All authors have read and approved its final version.
